# Safety and effectiveness of switching to Abacavir/Lamivudine plus rilpivirine for maintenance therapy in virologically suppressed HIV-1 individuals in Singapore (SEALS)

**DOI:** 10.1186/s12981-021-00402-7

**Published:** 2021-11-01

**Authors:** Z. C. Lim, G. S. Hoo, J. H. Ang, C. B. Teng, L. W. Ang, C. C. Lee, Y. S. Leo, H. L. Law, O. T. Ng, C. S. Wong

**Affiliations:** 1 Department of Pharmacy, Ng Teng Fong General Hospital, Singapore, Singapore; 2 Department of Pharmacy, National Centre of Infectious Diseases, Singapore, Singapore; 3 Department of Pharmacy, Tan Tock Seng Hospital, Singapore, Singapore; 4 Department of Pharmacy, National University of Singapore, Singapore, Singapore; 5 National Public Health and Epidemiology Unit, National Centre for Infectious Diseases, Singapore, Singapore; 6 National Centre for Infectious Diseases, Singapore, Singapore; 7 Department of Infectious Diseases, Tan Tock Seng Hospital, Singapore, Singapore; 8 Yong Loo Lin School of Medicine, National University of Singapore, Singapore, Singapore; 9 Lee Kong Chian School of Medicine, Nanyang Technological University, Singapore, Singapore; 10 Saw Swee Hock School of Public Health, National University of Singapore, Singapore, Singapore

**Keywords:** Maintenance, Switch therapy, Virologically suppressed, Abacavir, Rilpivirine, HIV

## Abstract

**Background:**

The efficacy and tolerability of an antiretroviral regimen are important considerations for selection of HIV-1 infection maintenance therapy. Abacavir/lamivudine plus rilpivirine (ABC/3TC + RPV) has been shown in international studies to be effective and well-tolerated in virologically suppressed individuals. This study evaluated the effectiveness and safety of switching to ABC/3TC + RPV as maintenance therapy in virologically suppressed HIV-1 infected individuals in Singapore.

**Methods:**

In this retrospective, single-centre study, we included individuals who were prescribed ABC/3TC + RPV, had HIV-1 viral load (VL) < 50 copies/ml immediately pre-switch, and had no documented history of resistance mutations or virologic failure to any of the components. The follow-up period was 48 ± 12 weeks. The primary outcome was the proportion of individuals who maintained virologic suppression of HIV-1 VL < 50 copies/ml at the end of follow-up period based on on-treatment analysis. The secondary outcomes were the resistance profiles associated with virologic failure, changes in immunologic and metabolic parameters, and the safety profile of ABC/3TC + RPV.

**Results:**

A total of 222 individuals were included in the study. The primary outcome was achieved in 197 individuals [88.8%, 95% confidence interval: 83.7–92.4%]. There were 21 individuals (9.5%) who discontinued treatment for non-virologic reasons. The remaining 4 individuals experienced virologic failure, of whom, 3 of these individuals had developed emergent antiretroviral resistance and had HIV-1 VL > 500 copies/ml at the end of the 48 ± 12 weeks follow-up period. The remaining individual experienced sustained low level viremia and subsequently achieved viral suppression without undergoing resistance testing. A total of 49 adverse events were observed in 31 out of 222 individuals (14.0%), which led to 13 individuals discontinuing therapy. Neuropsychiatric adverse events were most commonly observed (53.1%). A statistically significant increase in CD4 was observed (p < 0.01), with a median absolute change of 31 cells/uL (interquartile range: − 31.50 to 140.75). No significant changes in lipid profiles were detected.

**Conclusion:**

ABC/3TC + RPV is a safe and effective switch option for maintenance therapy in virologically suppressed HIV-1 individuals with in Singapore.

**Supplementary Information:**

The online version contains supplementary material available at 10.1186/s12981-021-00402-7.

## Background

Modern combination antiretroviral therapy (ART) regimens have generally demonstrated potency and efficacy in achieving virologic suppression [1]. Owing to the chronic nature of HIV infection, maintenance therapy is required even after initial virologic suppression [1]. In addition to efficacy, the cost-effectiveness, convenience and long-term tolerability of the ART are important considerations in selecting a suitable maintenance therapy [1].

The current standard for HIV treatment is combination ART, which comprise of two nucleos(t)ide reverse transcriptase inhibitors (NRTI) as backbone, paired with a third agent from another class, including integrase strand transfer inhibitors (INSTI), non-nucleoside reverse transcriptase inhibitors (NNRTI), or protease inhibitors (PI) [1]. Tenofovir and emtricitabine (TDF/FTC) are recommended as a first-line NRTI backbone for their effectiveness, but the combination of abacavir and lamivudine (ABC/3TC) may be used as maintenance therapy for its cost-effectiveness and better safety profile [1–4]. Compared to tenofovir-based regimens, ABC/3TC offer the advantage of avoiding potential renal and bone toxicity and is suitable for use in individuals with renal insufficiency and osteoporosis [1–4]. However, several studies suggest a possible increased cardiovascular risk for abacavir-based regimens, which remains a clinical concern [5, 6]. In Singapore, the price of ABC/3TC is roughly half that of TDF and 3TC (as separate components), and one-fifth the cost of a TDF/FTC fixed dose combination tablet. Rilpivirine (RPV) is a second-generation NNRTI approved for use with NRTIs in treatment-naïve HIV-1 infected individuals [7–11]. Its use is recommended to be limited to individuals with pre-treatment CD4 counts exceeding 200 cells/uL and pre-treatment viral loads of less than 100,000 copies/ml [1]. RPV is also contraindicated in individuals with concomitant proton pump inhibitor use, and requires to be administered with a normal- to high-calorie meal for optimal absorption. Nonetheless, RPV has been shown to have fewer neuropsychiatric adverse effects (AEs) when compared with efavirenz, a more favourable metabolic profile compared with PI, and better cost-effectiveness due to its relatively lower cost [7–11]. In Singapore, the cost of RPV is 25% compared to that of a PI or INSTI. Due to the lower cost of ABC/3TC and RPV, they remain as enticing options for selected individuals who qualify for its use.

The SIMRIKI retrospective study has demonstrated the long-term effectiveness and safety of ABC/3TC + RPV as a switch therapy with a follow-up period of up to 48 weeks [12]. The results of the SIMRIKI study are corroborated by several similar Spanish studies [13–15]. In particular, these international studies seem to reinforce the long-term cardiovascular safety of ABC/3TC + RPV [12–15]. A local study has also shown the efficacy and safety of this regimen in treatment naïve HIV-1 infected adults, [16] but to our knowledge, there have not been any studies on the effectiveness and safety of ABC/3TC + RPV as a switch regimen in Singapore.

The aim of this study was to evaluate the effectiveness and safety of switching to ABC/3TC + RPV as maintenance therapy for virologically suppressed HIV-1 infected individuals in Singapore.

## Methods

This study was a retrospective, single-centre evaluation conducted at National Centre for Infectious Diseases (NCID), which provides HIV care for the majority of HIV-infected individuals in Singapore. The study was conducted between June 2014 and September 2018. Approval from Domain Specific Review Board of the National Healthcare Group (Reference Number: 2012/00438-SRF0006) was obtained prior to study commencement.

The inclusion criteria were as follows: (i) documented HIV-1 infection, (ii) age ≥ 18 years, (iii) use of combination of ABC/3TC (600/300 mg fixed dose combination once daily) plus RPV (25 mg once daily) as maintenance therapy between June 2014 and September 2018 inclusive, (iv) virologically suppressed (defined as having at least 1 reading of HIV-1 viral load (VL) < 50 copies/ml) immediately before switching to ABC/3TC + RPV, and (v) no documented history of resistance mutations or virologic failure to ABC, 3TC, FTC or RPV. Exclusion criteria were CD4 count < 200 cells/uL and pregnancy. As adherence could not be determined for individuals who either obtained external supply of ART with no pharmacy dispensed records and/or with erratic clinic attendance, individuals with proportion of days covered (PDC) for adherence of < 95% were also excluded. PDC was calculated by the following formula: [Number of days with medication supply in study period / number of days in the study period] × 100, capped at 100%. PDC is a known, validated and stringent measure of adherence [17, 18]. The cut-off for PDC was chosen to be 95% as it is a widely used standard for optimal ART [19].

Eligible individuals were identified from the NCID HIV Clinical Database, a standardised computerised database containing records of demographic information, HIV transmission route, and ART history. Data collected from electronic medical records included AEs attributed to ART, reasons for ART switch, and other laboratory readings which were not available on the Clinical Database. Data were anonymised upon extraction. Intervals for follow-up were determined by individual attending physicians per in-house clinical care guidelines.

The primary outcome measured was the proportion of individuals who maintained virologic suppression on ABC/3TC + RPV after 48 ± 12 weeks of follow-up and had no therapy discontinuation due to non-virologic reasons such as adverse drug reactions. Individuals were considered to have maintained virologic suppression if they had no virologic failure. Virologic failure was defined as: (i) 2 consecutive HIV-1 VL readings of > 50 copies/ml, (ii) any HIV-1 VL > 50 copies/ml that resulted in discontinuation of ABC/3TC + RPV, or (iii) any HIV-1 VL reading of > 500 copies/ml. This definition for virological failure is similar to the SIMRIKI study, [12] but also included any HIV-1 VL reading of > 500 copies/ml to detect any potential emergent antiretroviral resistance. Individuals who had a one-off HIV-1 VL reading between 51 copies/ml and < 200 copies/ml that was followed by a return to virologic suppression were considered virologic blips [1]. For all individuals, HIV-1 VL was measured at least twice throughout the follow-up period.

The secondary outcomes were the resistance profiles associated with virologic failure, changes in immunologic and metabolic parameters, and the AE profile of ABC/3TC + RPV. For the immunologic and metabolic parameters, only individuals who had both baseline and follow-up values were analyzed for secondary outcomes.

The 95% confidence intervals (CI) were calculated based on the Newcombe-Wilson hybrid score [20]. We checked whether the continuous variables were normally distributed using the Kolmogorov–Smirnov test (for n ≥ 50) and Shapiro–Wilk test (for n < 50). We then used the paired samples t-test to analyze variables with normal distribution, and the Wilcoxon Signed Rank test to analyze variables with non-normal distribution. All statistical tests were two-sided, and p-values < 0.05 were considered statistically significant. Statistical analysis was carried out using the IBM SPSS Statistics for Windows, version 24.0 (Armonk, NY: IBM Corp).

## Results

**Fig. 1:**
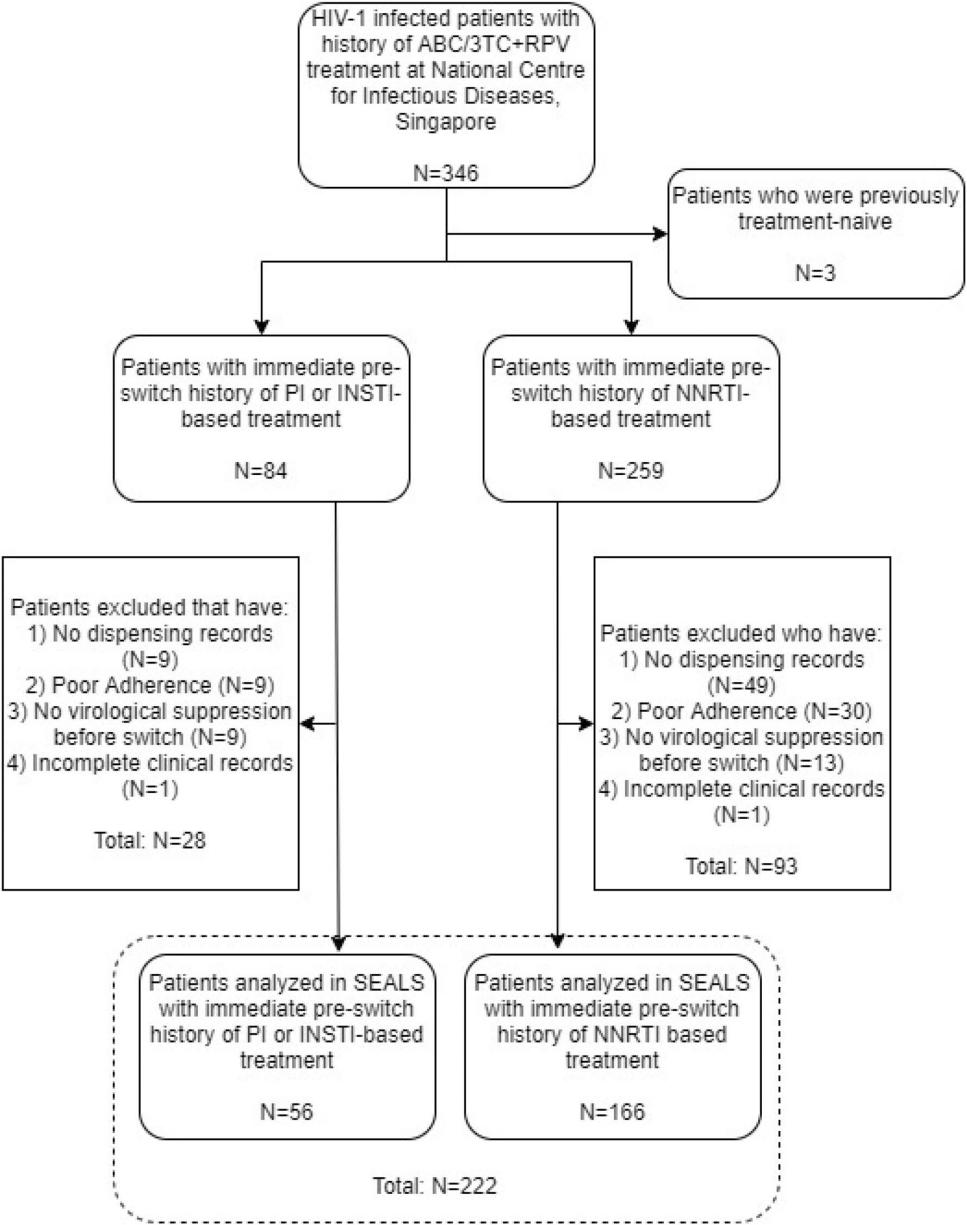
Inclusion Flowchart

We collected data from 346 individuals. A total of 222 individuals were included for analysis. The inclusion process of the study was detailed in Fig. 1. Baseline characteristics of the individuals included in the study were displayed in Table 1.

**Table 1:** Baseline characteristics of study population

Baseline Characteristic		
Age (years), median (IQR)		47 (35–57)
Male, n (%)		205 (92.3)
Duration of known HIV infection (years), median (IQR)		5.6 (3.6–8.4)
Race, n (%)		
Chinese		176 (79.3)
Malay		32 (14.4)
Indian		12 (5.4)
Others		2 (0.9)
HIV Risk Group, n (%)		
Heterosexual Contact Only		96 (43.2)
Homosexual Contact Only		93 (41.9)
Bisexual Contact Only		22 (9.9)
Intravenous Drug Use (IDU) Only		3 (1.4)
Others (Heterosexual Contact + IDU, Homosexual Contact + IDU, Bisexual Contact + IDU)		3 (1.4)
Unknown		5 (2.3)
Previous ART, n (%)		
Backbone		
Tenofovir/Lamivudine or Tenofovir/Emtricitabine		135 (60.8)
Abacavir/Lamivudine		60 (27.0)
Zidovudine/Lamivudine		23 (10.3)
Stavudine/Lamivudine		4 (1.9)
Third Agent		
Efavirenz		94 (42.3)
Rilpivirine		52 (23.4)
Atazanavir/ritonavir		30 (13.5)
Nevirapine		20 (9.0)
Raltegravir		15 (6.8)
Darunavir/ritonavir		5 (2.3)
Dolutegravir		4 (1.8)
Lopinavir/ritonavir		2 (0.9)
Reasons for switching to ABC/3TC + RPV, n (%)		
Intolerance		117 (52.7)
Cost reduction		66 (29.7)
Simplification of regimen		52 (23.4)
Drug-drug interaction		5 (2.3)
Others		3 (1.4)
Unknown		31 (14.0)
Comorbidities, n (%)		
Myocardial infarction		4 (1.8)
Ischemic heart disease		5 (2.3)
Other cardiovascular pathologies		7 (3.2)
Hypertension		39 (17.6)
Diabetes mellitus		18 (8.1)
Dyslipidemia		67 (30.2)
Kidney disease		15 (6.8)
Hepatitis B		7 (3.2)
Hepatitis C		16 (7.2)
No. of individuals with 5 or more medications (polypharmacy), n (%)		63 (28.4)
Current smokers, n (%)		41 (18.5)

*IQR* interquartile range

The median age was 47 years (interquartile range [IQR]:35–57). Majority of the individuals were men (92.3%). The composition of pre-switch NRTI backbone (in combination with either 3TC or FTC) was as follows: TDF (60.8%), ABC (27.0%) zidovudine (10.3%), and stavudine (1.9%). The most common pre-switch third agents were efavirenz (42.3%) among the NNRTI, atazanavir/ritonavir (13.5%) among the PI, and raltegravir (6.8%) among the INSTI. Fifty-two (23.4%) individuals already had RPV as their third agent prior to switch. The most common pre-switch combination was TDF + 3TC + RPV, which accounted for 45/222 (20.3%) of all pre-switch regimens. Intolerance (52.7%), cost (29.7%), and simplification of regimen (23.4%) were the most common reasons cited for switching regimen. Thirty-one individuals (14.0%) had no documented reason for switch.

The primary outcome was achieved in 197 out of 222 individuals [88.8%, 95% confidence interval (CI): 83.7–92.4%]. For patients with an immediate pre-switch history of PI or INSTI-based treatment, 47 out of 56 individuals [83.9%, 95% CI: 72.2–91.3%] achieved the primary outcome. For patients with an immediate pre-switch history of NNRTI-based treatment, 150 out of 166 individuals [90.4%, 95% CI: 84.9–94.0%] achieved the primary outcome.

Four individuals (1.8%) had virologic failure (Additional file 1: Table S1), while 21 individuals (9.5%) discontinued treatment for non-virologic reasons before 48 ± 12 weeks (Additional file 1: Table S2). Among those who achieved the primary outcome, 7 individuals had viral blips during the 48 ± 12 weeks follow-up period.

Of the 4 individuals who experienced virologic failure, 3 individuals had HIV-1 VL > 500 copies/ml at the end of the study period and developed emergent antiretroviral resistance. The first individual experienced virologic failure on the 52^th^ week of switching regimen. Resistance testing showed M184MIV, V108VI, E138EK, K238KN mutations. This individual reported no missed doses, but administered ART at irregular timings with inconsistent caloric intake. The second individual failed on the 45th week of ABC/3TC + RPV and had M184I, E138K, H221HY mutations. He estimated missing doses once a week, and having erratic meal consumption. The third individual experienced virologic failure within 17 weeks of switching regimen, with emerging resistance mutations consisting of L74LV, M184I, E138EG, Y181YCF, M230L. This individual claimed full adherence to his ART, including administration with meals. The last individual had 3 consecutive readings of low-level viremia (HIV-1 VL > 50 copies/ml but < 200 copies/ml). This individual later achieved virologic suppression without the need to change regimen after the study period.

Among the 21 individuals who discontinued treatment for non-virologic reasons, 13/21 (61.9%) were due to AEs attributed to ABC/3TC + RPV. The details of these AEs that led to discontinuation are reported in Additional file 1: Table S2. Of the remaining 8/21 (38.1%) individuals that discontinued treatment for non-virologic reasons, half were to avoid drug-drug interactions with other concomitant medications, and one-quarter were due to personal financial issues. A total of 49 AEs were observed in 31 out of 222 individuals (14.0%) during the follow-up period (Table 2). All the AEs resolved spontaneously or upon therapy discontinuation. The most common type of AE was neuro-psychiatric (e.g., forgetfulness, insomnia, mood changes, vivid dreams), comprising 26/49 (53.1%) of all AEs reported. Notably, there were no cardiovascular AEs reported for ABC/3TC + RPV in the study period. A detailed breakdown of the AEs observed can be seen in Additional file 1: Table S3.

**Table 2:** Summary of adverse events

	n
Summary of adverse effects	
Total number of adverse effects	49
Individuals with ≥ 1 adverse effects	31
Individuals who discontinued therapy due to adverse effects	13
Types of adverse effects	
Neuro-psychiatric (i.e. forgetfulness, insomnia, mood changes, vivid dreams)	26
Digestive (i.e. abdominal pain, stomach-ache)	11
Dermatological (i.e. rash)	4
Others (e.g. fatigue, lipodystrophy, gynecomastia)	8

Data obtained on changes in the selected laboratory values over the study duration are summarized in Table 3. A statistically significant increase in CD4 was observed (p < 0.01), with a median absolute change of 31 cells/uL (IQR: − 31.50 to + 140.75). There were no significant changes in fasting blood glucose and HbA1c after switching regimen, based on the small number of individuals analysed (11 individuals for fasting blood glucose and 8 individuals for HbA1c). There was a statistically significant change observed for eGFR (p < 0.01), with a median absolute change of − 4.24 ml/min/1.73m^2^ (IQR: − 14.06 to + 5.27), while no significant change was detected for eCrCl (p = 0.463), with a median absolute change of − 0.79 ml/min (IQR: − 8.81 to + 7.97). Additional details on the stratification of eGFR results at baseline and at the end of the follow up period are reported in Additional file 1: Table S4. No significant changes in lipid profiles were detected. A statistically significant decrease of − 6U/L (IQR: − 11 to 0) was seen for ALT.

**Table 3:** Laboratory values at baseline and at end of follow-up period

		Median (IQR)	p-value
Laboratory parameter			
Absolute CD4 Count (n = 38)	Baseline (cells/uL)	323 (220 to 519)	
	Follow up (cells/uL)	397 (252 to 603)	
	Follow up Duration (Weeks)	49 (44 to 52)	
	Change (cells/uL)	31 (− 32 to + 141)	< 0.01^b^
Glucose monitoring			
Fasting blood glucose (n = 11)	Baseline (mmol/L)	5.1 (4.9 to 5.7)	
	Follow Up (mmol/L)	5.2 (4.7 to 5.5)	
	Follow up Duration (Weeks)	50 (46 to 52)	
	Change (mmol/L)	0.3 (− 0.5 to + 0.4)	0.654^b^
HbA1c (n = 8)	Baseline (%)	5.75 (5.55 to 6.5)	
	Follow Up (%)	5.85 (5.5 to 6.78)	
	Follow up Duration (Weeks)	50 (40 to 58)	
	Absolute Change (%)	0.05 (− 0.08 to + 0.2)	0.288^a^
Renal			
eGFR (n = 139)	Baseline (ml/min/1.73m^2^)	90 (78 to 104)	
	Follow Up (ml/min/1.73m^2^)	87 (76 to 99)	
	Follow up Duration (Weeks)	49 (40 to 54)	
	Change (ml/min/1.73m^2^)	− 4 (− 14 to 5)	< 0.01^b^
eCrCl (n = 139)	Baseline (ml/min)	91 (76 to 106)	
	Follow Up (ml/min)	90 (76 to 106)	
	Follow up Duration (Weeks)	49 (40 to 54)	
	Change (ml/min)	− 1 (− 9 to + 8)	0.423^a^
Lipids			
TC (n = 38)	Baseline (mmol/L)	5.1 (4.6 to 6.0)	
	Follow Up (mmol/L)	5.2 (4.4 to 5.8)	
	Follow up Duration (Weeks)	51 (45 to 54)	
	Change (mmol/L)	0.1 (− 1.1 to + 0.8)	0.778^b^
HDL-C (n = 38)	Baseline (mmol/L)	1.0 (0.9 to 1.4)	
	Follow Up (mmol/L)	1.2 (0.9 to 1.3)	
	Follow up Duration (Weeks)	50 (45 to 54)	
	Change (mmol/L)	0 (− 0.2 to + 0.2)	0.762^b^
dC LDL-C (n = 38)	Baseline (mmol/L)	2.9 (2.6 to 3.6)	
	Follow Up (mmol/L)	2.8 (2.4 to 3.5)	
	Follow up Duration (Weeks)	51 (45 to 54)	
	Change (mmol/L)	− 0.1 (− 0.5 to + 0.5)	0.817^b^
LDL-C (n = 35)	Baseline (mmol/L)	3.1 (2.6 to 3.7)	
	Follow Up (mmol/L)	3.1 (2.7 to 3.7)	
	Follow up Duration (Weeks)	51 (45 to 54)	
	Change (mmol/L)	0.1 (− 0.6 to + 0.7)	0.972^b^
Liver			
ALT (n = 117)	Baseline (U/L)	28 (21 to 44.5)	
	Follow Up (U/L)	22 (16 to 30)	
	Follow up Duration (Weeks)	48 (41 to 52)	
	Change (U/L)	− 6 (− 11 to + 0)	< 0.001^a^

*eGFR* Estimated glomerular filtration rate, *eCrCl* Estimated creatinine clearance, *TC* Total cholesterol, *HDL-C* High density lipoprotein cholesterol, *dC LDL-C *Low density lipoprotein cholesterol estimated with the de Cordova equation.  [21], *LDL-C* Low density lipoprotein cholesterol, *ALT* Alanine transaminase

^a^Wilcoxon signed rank test

^b^Paired samples t-test

## Discussion

Our results showed that a high proportion of individuals (88.8%) who switched to ABC/3TC + RPV had maintained virologic suppression. The result is similar regardless of the type of pre-switch ART regimen. Virologic suppression was achieved in 83.9% of individuals with an immediate pre-switch history of PI or INSTI-based treatment, and 90.4% of individuals with an immediate pre-switch history of NNRTI-based treatment. In several studies in Spain, 86–88% of HIV-1 infected individuals maintained virologic suppression and avoided therapy discontinuation at 48 weeks or 12 months [13–15]. The SIMRIKI study also saw 91.2% of their study population achieving the same outcome at 48 weeks [12]. The findings of our study corroborated existing primary literature that individuals on treatment with ABC/3TC + RPV can achieve effective, sustained virologic suppression.

A local study found that 96% of treatment naïve HIV-1 infected individuals achieved virologic suppression at the end of 48 weeks of treatment with ABC/3TC + RPV, [16] which was comparable to that of our study in which 98.0% (197/201) of individuals who completed follow-up without therapy discontinuation remained virologically suppressed. A subgroup analysis regarding the type of pre-switch ART regimen for these individuals yielded similar results. 97.9% (47/48) of the individuals in the pre-switch PI or INSTI-based treatment group and 98.0% (150/153) of the individuals in the pre-switch NNRTI-based treatment group who completed follow-up without therapy discontinuation remained virologically suppressed. These results further echo the strongly positive existing literature on the effectiveness of ABC/3TC + RPV, especially when there is a high level of compliance. In the event of virologic failure to ABC/3TC + RPV, individuals still have the option of switching to a salvage regimen which includes a PI or INSTI or both.

AEs were observed amongst 31/222 individuals (14.0%) and resolved either spontaneously or upon discontinuation of the medication. These results were comparable to the favourable results of SIMRIKI study, [12] as well as the local study on treatment-naïve HIV-infected individuals, [16] which reported only 15.6% and 13.5% of individuals with observed AEs respectively. The AE profile seen in our study was also very similar to the local study which saw mostly neuropsychiatric AEs at a rate of 46.4% of all observed AEs compared to our study which saw 53.1% of observed AEs being neuropsychiatric [16]. In addition, none of the AEs observed were cardiovascular, in contrast to other studies that suggest the cardiovascular toxicity of ABC-based regimens [5, 6]. These findings further support the well-established safety and tolerability of ABC/3TC + RPV.

Our cohort had a significant improvement of absolute CD4 count at the end of the 48 ± 12 weeks follow-up. The increase in the CD4 count can possibly be attributed to maintained virologic suppression, and was also seen in other switch studies with increases ranging from + 48 to + 262 cells/uL [13–15].

We observed a statistically significant decrease in eGFR but not in eCrCl. The median absolute changes in eGFR and eCrCl were deemed to be of little or no clinical relevance [22, 23], as a median change of − 4 ml/min/1.73m^2^ in eGFR or − 1 ml/min in eCrCl is unlikely to affect the staging of chronic kidney disease or acute kidney injury. We also observed a statistically significant decrease in ALT, but the magnitude of the change is clinically insignificant, [24] as the median change of − 6 U/L is insufficient to warrant any clinical action. Significant changes in eCrCl, eGFR and ALTs were rarely noted in other studies on ABC/3TC + RPV, and it is likely that the regimen has no significant effect on these laboratory parameters [13–16].

While no statistically significant changes were detected for lipids, fasting blood glucose and HbA1c, the results warrant careful interpretation due to the small number of individuals with both baseline and follow-up values. Remarkably, the SIMRIKI study and the studies by Curran et al. and Palacios et al. all noted significant decreases in total cholesterol and LDL-C, as well as increases in HDL-C [12–14]. However, the local study by Ho et al. did not lend evidence to any significant effect of the regimen on lipids, [16] and there are no studies so far that we know of that have documented significant changes in fasting blood glucose and HbA1c.

Our study has several limitations. The absence of a control group prevented us from comparing the effectiveness and safety in individuals who would have continued with the prior regimen. We did not have access to baseline resistance testing information for our study population. As we excluded individuals who were < 95% adherent by PDC, our findings may not be generalizable to individuals with suboptimal adherence or who are compliant to ART despite irregular clinic attendance or who purchase external supplies of ART. Compliance to these medications will remain a key clinical prerequisite to therapy success. In practice, it will be crucial to take into account the relatively low genetic barrier to resistance and the ability of the individual to adhere to the caloric requirements of RPV when selecting this regimen. The frequency of adverse events may also have been under-reported, as only adverse events that were reported and explicitly attributed to the regimen in clinical documentation were included in the study.

In conclusion, this study shows that ABC/3TC + RPV is an effective and safe switch option for maintenance therapy in virologically suppressed HIV-1 individuals in Singapore.

## Supplementary Information

**Additional file 1: Table S1**. Details of 4 individuals who failed the primary outcome for virological reasons. **Table S2**. Details of 21 individuals who failed the primary outcome for non-virological reasons. **Table S3**. Breakdown of All AEs. **Table S4**. Changes in eGFR stratificationA from baseline to end of follow up period (n=139)

## Data Availability

The dataset generated and/or analysed during the current study are not publicly available due to the Personal Data Protection Act in Singapore and the sensitivity of the diagnosis, but reversibly anonymised dataset is available from the corresponding author on reasonable request.
