# Supplementary material for: Safety and effectiveness of switching to Abacavir/Lamivudine plus rilpivirine for maintenance therapy in virologically suppressed HIV-1 individuals in Singapore (SEALS)
Source: AIDS Res Ther. 2021 Nov 1;18:80. doi: 10.1186/s12981-021-00402-7 (PMC8561921; doi:10.1186/s12981-021-00402-7)
Supplement: Supplementary file 1 — Additional file 1: Table S1. Details of 4 individuals who failed the primary outcome for virological reasons. Table S2. Details of 21 individuals who failed the primary outcome for non-virological reasons. Table S3. Breakdown of All AEs. Table S4. Changes in eGFR stratificationA from baseline to end of follow up period (n=139) [file 12981_2021_402_MOESM1_ESM.docx]

**Appendix**

**Supplementary Table 1: Details of 4 individuals who failed the primary outcome for virological reasons**

| **No** | **Consecutive VL >50 readings during study period** | **VL >50 at the end of study period** | **VL >500 during study period** | **cART change during study period** | **Subsequent discontinuation of cART after study period** |
| --- | --- | --- | --- | --- | --- |
| 1 | Yes | Yes | No | No | No |
| 2 | No | Yes | Yes | No | Yes |
| 3 | No | Yes | Yes | No | Yes |
| 4 | Yes | Yes | Yes | No | Yes |
| VL, Viral Load (copies/ml) | | | | |  |

**Supplementary Table 2: Details of 21 individuals who failed the primary outcome for non-virological reasons**

| **No.** | **Reason** | **Reasons for discontinuation** | | **Remarks** |
| --- | --- | --- | --- | --- |
|  |  | **AE** | **Non-AE** |  |
| 1 | Switch Therapy | Yes | No | Lethargy, Abdominal Pain (ABC/3TC/RPV) |
| 2 | Switch Therapy | Yes | No | Lipodystrophy, Gynecomastia (ABC/3TC) |
| 3 | Switch Therapy | Yes | No | Unspecified Pain (ABC/3TC) |
| 4 | Switch Therapy | Yes | No | Headaches, Stomachaches (ABC/3TC) |
| 5 | Switch Therapy | Yes | No | Rash (ABC/3TC) |
| 6 | Switch Therapy | Yes | No | Fatigue (RPV) |
| 7 | Switch Therapy | Yes | No | Systemic Adverse Effects (RPV) |
| 8 | Switch Therapy | Yes | No | Eye Swelling, Dizziness, Malaise (RPV) |
| 9 | Switch Therapy | Yes | No | CNS Effects, Drowsiness, Forgetfulness (RPV) |
| 10 | Switch Therapy | Yes | No | Non-Vertiginous Giddiness (RPV) |
| 11 | Switch Therapy | Yes | No | Mood Issues, Insomnia, Vivid Dreams (RPV) |
| 12 | Switch Therapy | Yes | No | Mood Issues (RPV) |
| 13 | Switch Therapy | Yes | No | Malaise (Undetermined Cause) |
| 14 | Switch Therapy | No | Yes | Administration Difficulties (RPV) |
| 15 | Switch Therapy | No | Yes | Drug-Drug Interaction |
| 16 | Switch Therapy | No | Yes | Drug-Drug Interaction |
| 17 | Switch Therapy | No | Yes | Drug-Drug Interaction |
| 18 | Switch Therapy | No | Yes | Drug-Drug Interaction |
| 19 | Switch Therapy | No | Yes | Switched out to improve barrier to resistance |
| 20 | Switch Therapy | No | Yes | Personal Financial Issues |
| 21 | Restarted Therapy | No | Yes | Personal Financial Issues |

AE, adverse event

**Supplementary Table 3: Breakdown of All AEs**

| **Case Number** | **Neuropsychiatric** | **Gastro-intestinal** | **Dermatological** | **Others** | **Discontinued due to AE?** |
| --- | --- | --- | --- | --- | --- |
| 1 | Sleepiness | Abdominal Pain |  |  | Yes |
| 2 |  |  |  | Lipodystrophy (Gynecomastia) | Yes |
| 3 |  |  |  | Unspecified Pain | Yes |
| 4 | Headache | Stomach-ache |  |  | Yes |
| 5 |  |  | Rash |  | Yes |
| 6 |  |  |  | Fatigue | Yes |
| 7 | Drowsiness |  |  | Fatigue | Yes |
| 8 | Dizziness |  |  | Eye Swelling, Malaise | Yes |
| 9 | Drowsiness, Forgetfulness |  |  |  | Yes |
| 10 | Giddiness |  |  |  | Yes |
| 11 | Low Mood, Insomnia, Vivid Dreams |  |  |  | Yes |
| 12 | Low Mood |  |  |  | Yes |
| 13 |  |  |  | Malaise | Yes |
| 14 | Dizziness |  |  |  | No |
| 15 | Giddiness |  |  |  | No |
| 16 | Drowsiness |  |  |  | No |
| 17 | Insomnia |  |  |  | No |
| 18 | Drowsiness, Headache |  |  |  | No |
| 19 | Vivid Dreams |  |  |  | No |
| 20 | Insomnia |  |  |  | No |
| 21 | Low mood, Insomnia |  |  |  | No |
| 22 | Mood Swings |  |  |  | No |
| 23 | Headache, Giddiness | Abdominal Pain, Nausea | Itch |  | No |
| 24 |  |  | Itch, Flushing |  | No |
| 25 |  | Bloatedness, Heartburn |  |  | No |
| 26 |  | Nausea |  |  | No |
| 27 |  | Nausea |  |  | No |
| 28 |  | Nausea, Diarrhea |  |  | No |
| 29 | Low Mood |  |  |  | No |
| 30 | Low Mood |  |  |  | No |
| 31 |  | Bloatedness |  | Dehydration | No |

AE, adverse event

**Supplementary Table 4: Changes in eGFR stratification^A^ from baseline to end of follow up period (n=139)**

| **At baseline, patients in KDIGO GFR category^A^:** | **Number of patients (%)** |
| --- | --- |
| - G1 | 70 (50.4) |
| - G2 | 60 (43.2) |
| - G3a | 6 (4.3) |
| - G3b | 2 (1.4) |
| - G4 | 1 (0.7) |
| **At the end of the follow up period, patients in KDIGO GFR category^22^:** | **Number of patients (%)** |
| - G1 | 59 (42.4) |
| - G2 | 65 (46.8) |
| - G3a | 10 (7.2) |
| - G3b | 5 (3.6) |

eGFR, estimated glomerular filtration rate

^A^ Derived from the KDIGO 2012 Clinical Practice Guideline for the Evaluation and Management of Chronic Kidney Disease. Retrieved from https://kdigo.org/wp-content/uploads/2017/02/KDIGO_2012_CKD_GL.pdf. Published January 2013.
